# Supplementary material for: Impact of work competencies on job performance among university counsellors
Source: PLoS One. 2024 Dec 11;19(12):e0315494. doi: 10.1371/journal.pone.0315494 (PMC11634011; doi:10.1371/journal.pone.0315494)
Supplement: Attachment — Submitted filename: Response to reviewers. docx.docx [file pone.0315494.s002.docx]

**Dear Editor and Reviewers,**

Thank you for your valuable comments regarding our manuscript. We appreciate your insights, which have been instrumental in revising and enhancing our paper. We have carefully considered each point and made the necessary corrections. Below are our detailed responses to the reviewers’ comments:

**Reviewer 1:**

**Reviewer point 1:** Add sampling characteristics also (you can add a table of demographic characteristics of sample, e.g. Age, gender, education, experience etc).

**Author response 1:** The authors add the table 1 to show the demographic information of this research.

**Reviewer point 2:** Clearly state the Spencer and Spencer description of the competency theory factors.

**Author response 2:** The authors added more statements about the competency theory factors in theoretical foundation.

**Reviewer point 3:** The need and process used for adaptation should be added before statistical models or analyses.

**Author response 3:** The author added the justification and process of choosing the statistical analysis method.

**Reviewer point 4:** Add a more detailed exploration of the implications of its non-significant findings.

**Author response 4:** The author added more explanation of the non-significant finding.

**Reviewer point 5:** Add more critical discussion of its limitations.

**Author response 5:** The author added more discussion of limitation part.

**Reviewer 2:**

**Reviewer point 1:** The document need editing corrections. There are many examples where vague and overly long sentences are used, leading to a lack of clarity.

**Author response 1:** The authors revised the full paper and finished the proofreading.

**Reviewer point 2:** The work competency factors are not operationally defined, which hinders the reader’s understanding of how these constructs are measured.

**Author response 2:** The definitions of work competency factors are added in the theoretical foundation section and in the literature review section.

**Reviewer point 3:** The tools used for data collection are not adequately discussed in the methodology section, that is critical to discuss.

**Author response 3:** The authors revised this part and added more statement about the

tools.

**Reviewer point 4:** The discussion seems to be focused on the conflicting roles of counsellors, which detracts from addressing the actual discussion according to the hypotheses.

**Author response 4:** To avoid the misunderstanding of this part, the authors revised the conflicting role and added more discussion about the result.

**Reviewer point 5:** Additionally, the suggestions for administrators and policymakers presented in the paper are overly broad. More specific recommendations would enhance the practical implications of the study.

**Author response 5:** The authors added more specific recommendations in the managerial implication part.

**Reviewer point 6:** Similarly, under the conclusion section, the suggestions are repeated. It would be more effective to suggest concrete and actionable recommendations.

**Author response 6:** To avoid the repetition, the authors put the suggestion part inside the managerial implication part and give more specific recommendations.
